# Targeted immunotherapy for hair regrowth and regeneration

**DOI:** 10.3389/fmed.2023.1285452

**Published:** 2023-10-10

**Authors:** En Qi Toh, Etienne C. E. Wang

**Affiliations:** ^1^Lee Kong Chian School of Medicine, Nanyang Technological University, Singapore, Singapore; ^2^National Skin Centre, Singapore, Singapore

**Keywords:** targeted, immunotherapy, hair, alopecia, regrowth, regeneration

## 1. Introduction

Immunotherapy for skin conditions has a long and successful history. While the main strategy for treating inflammatory conditions is local or systemic immunosuppression, immunotherapy aims to stimulate parts or all of the immune system to bring about a therapeutic response. The immune system contains multiple, redundant avenues of checks and balances, and some immune cells have immunomodulatory or regulatory roles. These cells are targeted by immunotherapy in inflammatory skin conditions. Immunotherapy is effective in treating some hair loss disorders, supporting recent findings that the hair cycle is influenced by immune cells (1, 2).

The normal hair cycle consists of sequential growth (anagen), regression (catagen) and rest (telogen) phases. In humans, the anagen phase (9–10 years) reflects the growing hair shaft, while the end of telogen (2–3 months) is marked by hair shedding. When the hair cycle is disrupted, premature anagen-to-catagen transition results in majority of hair follicles (HFs) entering telogen and shedding concurrently, causing significant, noticeable hair loss.

The immune system is closely associated with the HF, with macrophages and mast cells described in the perifollicular dermis (3, 4). Skin-resident macrophages clear apoptotic cell fragments following catagen (5) and may also interact with hair follicle stem cells (HFSC) to maintain quiescence during telogen (1). The dynamic nature of the hair cycle results in a constant flux of antigens, both self and non-self, from the manufacture of the hair shaft and the infundibular connection to the outside world, respectively. To protect the hair bulb from inappropriate immune attack, the HF has evolved a status of relative “immune privilege” (IP). This was demonstrated elegantly by Billingham et al. decades ago showing that the presence of HFs in allografts was sufficient to prevent immune rejection of melanocytes in transplantation experiments on guinea pigs (6). Various mechanisms have since been proposed for the IP of the HF, with the most prominent one being a downregulation of MHC Class I expression (7). Hair loss disorders are associated with disruptions and changes in the immune milieu of the HF (8, 9), and conversely, immunotherapy has been utilized to promote HF regrowth and regeneration.

## 2. The immune system interaction with the hair follicle niche

With evidence of immune cells being associated with the HF, it is reasonable to hypothesize that changes in immune cell composition and activity around HFs can affect HFSC and the hair cycle. However, the direct influence of immune cells in promoting hair regrowth and regeneration in humans is still unknown. In mice, regulatory T-cells (T-regs) promote anagen re-entry via Notch signaling (10), and macrophages may release Wnt factors to stimulate anagen under certain conditions (11). Wound-induced HF neogenesis (WIHN) is another phenomenon in rodents whereby brand-new HFs arise from scar tissue of a wound. Cotsarelis and Ito showed that relatively large wounds in the back skin of mice initially heal without HFs, but these scars are soon populated by *de novo* HFs via WIHN (12). Small cutaneous wounds in mice upregulate Wnt and Shh pathways (13), while larger wounds recruit dermal γδ-T-cells (14) and M2 macrophages (15) to promote WIHN. While the mouse's fur coat and human scalp hair differ in their stage of hair cycle, fundamental processes and pathways are likely conserved, and human HFs may respond in a similar manner to microtrauma.

In humans, crude immune stimulation has been shown to induce hair growth. Friction and irritation are known causes of hypertrichosis, and excessive hair growth occurs after limb fixation with plaster cast application (16, 17), and on burned skin borders (18). Allergic contact dermatitis to wig adhesives is reported to have a therapeutic effect in alopecia areata (AA) (19), forming the basis of topical immunotherapy for AA. Contact dermatitis introduces “antigenic competition” which recruits suppressor T-cells and macrophages, producing an immunomodulatory environment and dampening the autoimmune attack on HFs (20). These reports point to a role for microtrauma to elicit a permissive environment for hair regrowth and regeneration.

Newer treatment modalities for AGA harnessing the effects of microtrauma include platelet-rich plasma (PRP), microneedling and ablative and non-ablative lasers, which aim to reproduce the effects of wounding in areas of alopecia. In PRP, intra-dermal injection of activated platelets release growth factors near HFs (21), including transforming growth factor-β (TGF-β), epidermal growth factor, basic fibroblast growth factor (FGF), vascular endothelial growth factor (VEGF), platelet-derived growth factor (PDGF), and insulin-like growth factor-1, which support the anagen phase of the hair cycle. The efficacy of PRP appears operator- and protocol-dependent, but has been shown in systematic reviews to promote hair regrowth modestly (22). In randomized, split-scalp studies of PRP vs. sham injections, hair density also increased among the controls, suggesting microtrauma alone may stimulate hair regrowth (23). Indeed, several groups have demonstrated that microneedling may be effective in treating AGA (24) and AA (25). Both ablative and non-ablative settings of Er:YAG lasers have been used in AGA and AA, with promising results (26–28).

The efficacy of these treatments supports the hypothesis that the microenvironment of HFSC can be manipulated to promote hair regrowth. Immune cells are likely involved in the process of microtrauma. The treatments above, however, are non-specific. They induce an irritant, allergic or wounding response that varies between patients, resulting in inconsistent effectiveness. We are still in the process of understanding this process, and fine tuning the procedures and patient selection to obtain the best possible clinical outcomes.

## 3. Targeted immunotherapy for hair regrowth

Targeted immunotherapy in promoting hair regeneration is expected to grow with understanding of the detailed control of the human hair cycle. The first mode of targeted immunotherapy has been applied for AA, as we know most about its immunopathology.

### 3.1. Immunosuppressive therapies with stimulatory action in other parts of the immune system or HFSC

#### 3.1.1. JAK inhibitors

Janus kinase (JAK) inhibitors have revolutionized therapeutics in dermatology, successfully utilized in many inflammatory skin conditions including atopic dermatitis. The JAK-STAT pathway is an integral component of AA pathophysiology, downstream of interferon-gamma (IFN-γ) and interleukin (IL)-15 signaling.

Baricitinib is the first FDA-approved drug for AA in 2022. In two concurrent phase III randomized controlled trials (BRAVE-AA1 and BRAVE-AA2), baricitinib achieved SALT scores <20 at 52 weeks with a good safety profile (29). Baricitinib, which inhibits JAK1 and JAK2 signaling is effective in long-standing AA resistant to traditional therapies (30), besides being safe and effective in pediatric AA (31). Increasingly specific JAK inhibitors (JAK1 ivarmacitinib, JAK3 ritlecitinib, and JAK1/Tyk2 beprocitinib) (29, 32) are investigated for use in AA, making treatment more targeted.

Topical JAK inhibition not only reverses hair loss in AA mice, but also induces the telogen-to-anagen transition in disease-free C56Bl/6 mice (33). This suggests that the JAK-STAT pathway is also involved in normal hair cycles (34), leading to the discovery of a distinct subset of TREM2+ macrophages that maintain HFSC quiescence by secreting oncostatin M (1). Pharmacological, immunological and genetic inhibition of these macrophages sufficiently induced anagen in mice. Whether a similar mechanism is present in the human HF niche is unknown, but STAT3 is upregulated in AGA scalps (35).

The Wnt/β-catenin signaling pathway is the major pathway in activating DPCs, which are crucial in the hair bulb and bulge interaction for anagen initiation. Treatment of HFs with ruxolitinib, a JAK1/2 inhibitor, stimulates the expression of β-catenin mRNA, upregulating Wnt/β-catenin signaling. Further, proinflammatory cytokines of AA, namely IFN-γ-induced caspase-1, IL-1β, IL-15 and IL-18 are also suppressed by JAK1/2 inhibition (36). JAK inhibition in AA thus may have more than an immunosuppressive role, and may have immunotherapeutic roles in stimulation of DP and/or HFSC.

In IFN-treated dermal papilla (DP) culture, ruxolitinib was also shown to downregulate MHC class I expression, contributing to partial IP restoration. In addition, ruxolitinib stimulated several growth factors, including FGF7, that supported DP cell survival which translates to a hair growth-permissive microenviroment independent of its immunosuppressive properties (36).

The *in vivo* effects of JAK inhibition have yet to be evaluated, partly due to the challenges in analyzing and quantifying the hair cycle, as follicular units are asynchronous and hair cycle phases last months to years. Existing JAK inhibitors also penetrate skin insufficiently (37).

Other challenges to JAK inhibition include disease recurrence after discontinuation and balancing long-term usage against side effects of infections, marrow suppression, transaminitis, and lipid abnormalities (38).

#### 3.1.2. Statins

Statins have been proposed for treating various dermatologic conditions characterized by ingress of activated leukocytes into the skin, including AA (39). In a pilot study, a combination of simvastatin and ezetimibe reduced hair loss and resulted in stable remission in AA mice model, with an increase in FOXP3+ T-regs (40). Simvastatin may improve AA through multidirectional pro- and anti-inflammatory activities. These include increasing Th2 cytokine secretion, upregulating T-reg cells in mice (39), downregulating Th1 cytokine secretion via JAK-STAT pathway modulation (41), inhibiting leukocyte activation, adhesion and migration (42), activating Wnt/β-catenin signaling pathway (43), and downregulating reactive oxygen species production (44). However, larger placebo-controlled trials are still required, as these findings were not always reproducible by other authors (45).

### 3.2. Biologic therapies

Monoclonal antibody therapy is not as well established for AA as for dermatological conditions like psoriasis and eczema. Most TNF inhibitors are ineffective for AA, while dupilumab was found to be modestly effective in a Phase IIa trial (46). Although AA is associated with atopic dermatitis and psoriasis, treatment for the latter does not always improve AA (47), and may sometimes worsen hair loss (48).

The contrasting effects of biological therapies and JAK inhibitors in AA suggest that current targets of monoclonal antibodies (TNF-α, IL-17, IL-23 and IL-4 signaling) may support the HF immune privileged microenvironment, and an unbalanced blockade of these pathways leads to cytokine imbalance and AA (49). JAK-inhibition is more focused on immune mechanisms that share JAK-STAT pathways, which are more frequently associated with “active” T-cell directives on the HF.

Nivolumab is an anti-PD1 (programmed cell death-1) monoclonal antibody effective in many cancers, including melanoma. It releases the inhibition of autoreactive T-cells, allowing immune system clearance of tumor cells. This same mechanism has been reported to cause AA in these patients (50). The PD-1/PD-L1 pathway has also been implicated in T-cell exhaustion accompanying response to AA treatment with JAK-inhibitors (51). Exploring this pathway in AA may lead to new targets for biological therapy.

### 3.3. IL-2 complex treatment

A pilot study using low dose IL-2 to expand T-reg populations in severe AA showed initial promise (52). T-regs suppress autoreactive NKG2D+ T-cells that attack HFs and promote hair regrowth by inducing anagen. In mice models, intradermal injection of IL-2/anti-IL-2 antibody complex (IL-2c) efficiently stimulates T-reg proliferation by 8- to 10-fold in the skin. T-regs have also been shown to promote anagen via Jagged-1 expression (10).

A prospective randomized control study with low dose IL-2 was conducted which showed limited efficacy of this treatment (53). In murine models, while the fold ratio of CD8 T-cells over T-regs was also markedly reduced post-IL-2c treatment, CD8 T-cells remained around HFs, including NKG2D+ T-cells, in established AA mouse models (54). Despite no significant reduction in IL-10 or TGF-β secretion, the expanded T-regs were not sufficient to inhibit CD8 T-cell proliferation in established AA, resulting in neither anagen induction nor AA reversal. Further studies are warranted to investigate the role of IL-2/IL-2c in the treatment of AA. Early IL-2c therapy has been hypothesized to be effective in acute AA, which may slow disease progression but may require adjunctive treatments for more chronic, established cases.

### 3.4. Prevention or restoration of IP collapse

AA develops when the IP of the HF collapses, due to ectopic MHC class-1 expression induced by IFN-γ (55). *Ex vivo-* application of TGF-β1, α-MSH and the drug FK506 (Tacrolimus) have been found to suppress MHC Class I expression in cultured HF organ cultures, likely through suppressing mRNA transcription (56). α-MSH, which also has immunosuppressive properties, is also increased in AA lesional scalp post-UVA phototherapy (57). While systemic calcineurin inhibitors like ciclosporin have been effective in treating severe recalcitrant AA, topical tacrolimus/FK506 has proven to be less reliable (58), and exploring this method of restoring HF IP may further expand our immunotherapy repertoire.

### 3.5. Harnessing microtrauma

For AGA, there is currently a wide variety of modalities for inducing microtrauma to promote hair regrowth, including fractional lasers and microneedling. While these may be non-targeted, they enable more targeted drug delivery when combined with topical treatments like minoxidil or PRP. Release of growth factors with microtrauma [such as PDGF, VEGF, β-catenin, Wnt3a and Wnt10b (59)] has been postulated to lead to angiogenesis, dermal thickening, adipogenesis and HF stem cell activation to promote anagen.

Identifying the key factors and signaling pathways that promote microtrauma-induced hair regrowth will pave the way to the next phase of targeted immunotherapy to treat hair loss. Inclusion of these growth factors, or PRP, into customized microneedles is currently explored as a delivery method for treating AGA (60). These factors, as well as other signaling molecules like nucleic acids, membrane receptors or co-factors, can be packaged in exosomes to deliver a targeted signal of hair regeneration (61, 62).

## 4. Conclusion

**Figure 1:**
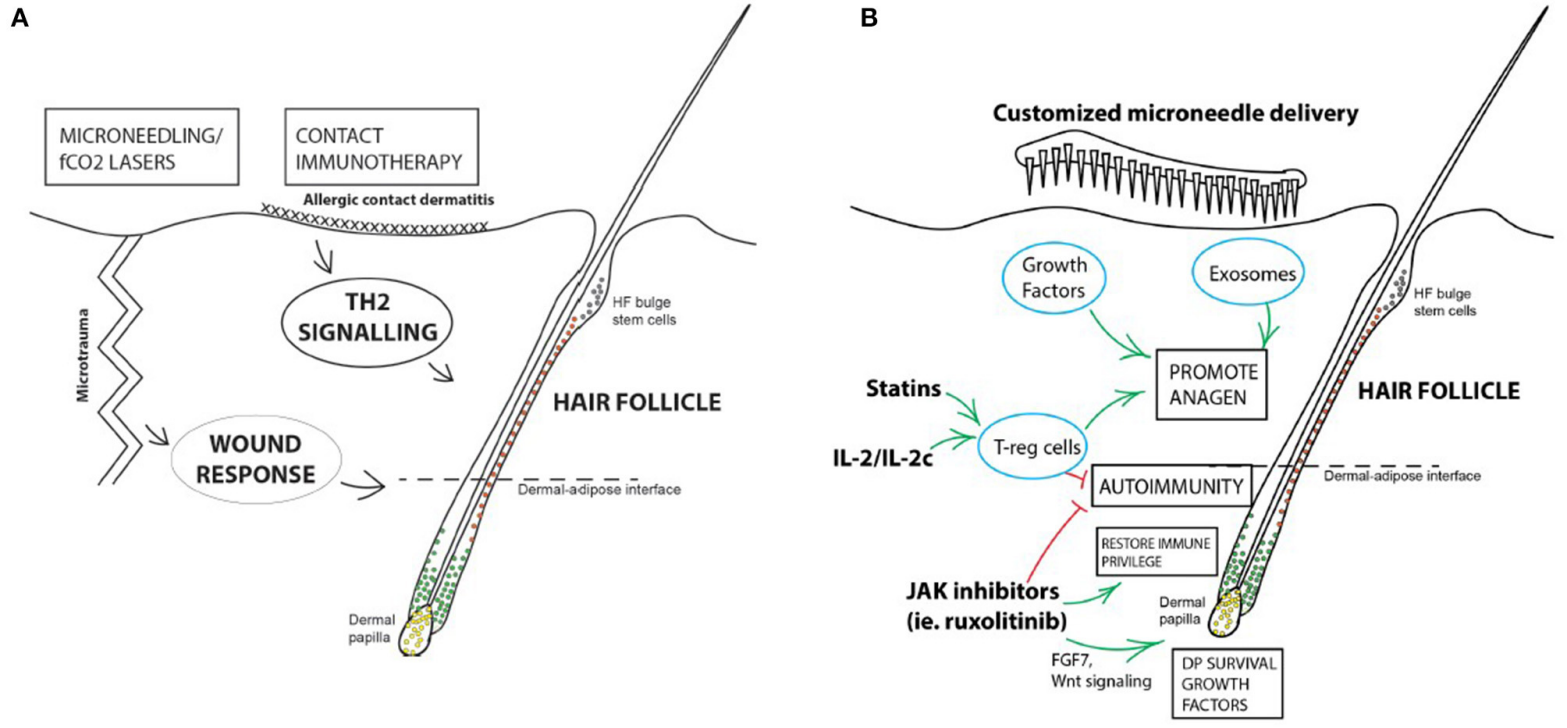
Summary of immunotherapy for hair loss disorders. **(A)** Current forms of immunotherapy are crude and non-targeted. Microtrauma produced by microneedling or PRP treatments are effective in promoting HF regeneration in AGA, and topical immunotherapy causing allergic contact dermatitis is an established treatment for AA. **(B)** Potential avenues for developing targeted immunotherapy for hair loss disorders. Some immunosuppressive treatments may have mechanisms of action independent of their suppressive effects, or direct stimulatory roles on the HF itself, like JAK inhibitors. JAK inhibition may have immunotherapeutic roles on the HF via upregulating the Wnt- β-catenin signaling pathway in DPCs, increasing FGF7 release which supports DP survival, and downregulation of MHC Class I expression in the HF, partially restoring IP. Stimulation of T-regs in the vicinity of the HF has both an immunomodulatory, as well as a direct pro-anagen effect on the HF. Targeted factors and signaling molecules can be packaged and delivered with bespoke microneedle patches or systems to mimic the effects of microtrauma to stimulate HF regrowth and regeneration.

Targeted immunotherapy (Figure 1) is a promising form of therapy for hair regrowth and regeneration, targeting immune cells that support and influence the hair cycle. Refining our current methods of immunotherapy will make these treatments more accessible to a wider population of patients suffering hair loss, with potentially fewer side effects. Further studies and controlled trials are required before they can be incorporated into clinical practice. If successful, targeted immunotherapy will provide hope for patients struggling with or have failed traditional treatments.

## Author contributions

ET: Writing—original draft, Writing—review and editing. EW: Conceptualization, Supervision, Writing—original draft, Writing—review and editing.
